# Effect of Hydroalcoholic and Buthanolic Extract of *Cucumis sativus *Seeds on Blood Glucose Level of Normal and Streptozotocin-Induced Diabetic Rats

**Published:** 2011

**Authors:** Mohsen Minaiyan, Behzad Zolfaghari, Amin Kamal

**Affiliations:** 1 *Department of Pharmacology & Toxicology, Isfahan Pharmaceutical Sciences Research Center, School of Pharmacy and Pharmaceutical Sciences, Isfahan University of Medical Sciences, Isfahan, Iran*; 2 *Department of Pharmacognosy, School of Pharmacy and Pharmaceutical Sciences, Isfahan University of Medical Sciences, Isfahan, Iran*

**Keywords:** Animal model, Blood Glucose, Diabetes mellitus, Hypoglycemic agents, Plant extract

## Abstract

**Objective(s):**

Seed of* Cucumis sativus* Linn. is one of the herbal remedies has been traditionally used for diabetes mellitus treatment. We studied the effect of hydroalcoholic and buthanolic extract obtained from *C. sativus* seeds in a model of streptozotocin (STZ)-induced diabetic (type I) rats.

**Materials and Methods:**

Normal and diabetic male Wistar rats (STZ, 60 mg/kg, intraperitoneal) were treated daily with vehicle (5 ml/kg), hydroalcoholic (0.2, 0.4, 0.8 g/kg) and buthanolic extract (0.2, 0.4, 0.8 g/kg) and glibenclamide (1 & 3 mg/kg) separately and treatment was continued for 9 days. Blood samples were taken at 0, 1, 2, 3, 4, 8 hr of the first day and the day 9 (216 hr) of treatments for measuring the blood glucose levels.

**Results:**

Our findings indicated that *C. sativus* seeds extracts were not effective on reducing blood glucose levels (BGL) in normal and diabetic rats for initial phase of treatments. However, both hydroalcoholic (22.5-33.8 %) and buthanolic (26.6- 45.0 %) extracts were effective on diminishing BGL and controling the loss of body weight in diabetic rats compared to controls after 9 days of continued daily therapy. Glibenclamide on the other hand, had hypoglycemic action in normal (27.8-31.0 %) and diabetic rats (36.0-50.0 %) after acute and prolonged treatments.

**Conclusion:**

It is concluded that *C. sativus* seeds extracts (hydroalcoholic and buthanolic) had a role in diabetes control probably through a mechanism similar to euglycemic agents. Further studies are warranted to clarify the mechanisms and the exact role of this herbal medicine in control of metabolic disorders.

## Introduction

Diabetes mellitus is a serious metabolic disease with several micro and macrovascular complications and diabetic patients have been rapidly increasing in number worldwide (1). There are currently about 170 million people worldwide with established diabetes mellitus and this figure is predicted to rise tremendously to more than 360 million till 2030 (2). In Iran, approximately 2 million people out of 75 million population suffer from diabetes mellitus and it is assumed that about 4.5 million people have impaired fasting glucose (3). An increase in ageing population, consumption of diets rich in fat and calories, sedentary life style and obesity are among the common risk factors and prevention of disease advancement will be a major concern in the 21^st^ century. It is obvious that the prevention of disease complications is possible by the control of blood glucose and improvement of hyperglycemia (4).

In addition to restriction of energy intake and exercise promotion, the usefulness of functional foods and herbal medicines during the daily life has been shown. For this purpose, several studies on functional foods and medicinal plants as well as their active components have been carried out to ascertain their usefulness in controlling the diabetes and their complications (5-7).

*Cucumis sativus* Linn. belongs to the Cucurbitaceae family and is widely distributed in the entire world especially in Asia, Africa and South America (7). Some Cucurbitaceae species have unique bitter taste and have been used in folk medicine for a long time (8). For example in Asian traditional medicine, *C. ficifolia*, popularly known as pumpkin is widely used for the treatment of diabetes mellitus (9,10). Another plant is *Momordical charantia *(family, Cucurbitaceae) which commonly known as Karela or bitter melon and is used as ethnomedicine for diabetes in India, China and South America (11,12). Besides, an investigation was carried out on three vegetable peels from Cucurbitaceae family including *C. pepo*, *C. sativus* and *Praecitrullus fistulosus* which revealed that they were all effective for prevention of alloxan-induced hyperglycemia in male mice (13). 

Main chemical constituents in Cucurbitaceae family are: volatile and fixed oils, saponins, steroids, carotenes, flavones, amino acids, resins, tannins, proteins and proteolytic enzymes for which several bioactivities like glucose and lipid lowering effects, diuretic, laxative, demulcent and anti-helminthic actions have been shown (7, 14-16). 

Seeds of *C. sativus* (*Tokhm-e-khiyar* in Persian) have been used as anti-fever, demulcent and antidiabetic in Iranian Traditional Medicine (17,18). Moreover functional studies have confirmed the hypoglycemic effect of *C. sativus* fruit extract in animal model of diabetes, however the probable effects of the plant seeds and its active constituents have not been elucidated yet (19,20). In the recent study, we investigated the effect of *C. sativus* seeds, using two extracts with different polarity to detect the probable anti-hyperglycemic or hypoglycemic activity. 

For this purpose, increasing doses of hydroalcoholic and buthanolic extracts of the plant seeds were examined in streptozotocin (STZ)-induced diabetic rats in comparison to glibenclamide as reference drug.

## Materials and Methods

***Plant material***

?^8^). All the reagents and organic solvents were purchased from Merck Company (Germany). The hydroalcoholic and butanolic extracts of C. sativus were screened for the presence of secondary metabolites e.g. anthraquinones, tannins, steroids, alkaloids and flavonoids according to the standard phytochemical methods (21).

The resulted extracts were then pooled, concentrated and dried using the rotary and freeze dryer apparatus respectively. The yield values of 8.9% and 3.3% were obtained for hydroalcoholic and buthanolic extracts respectively.

***Animals***

Male Wistar rats, 4 months old (200-250 g) were obtained from the animal house of School of Pharmacy, Isfahan University of Medical Sciences and maintained under uniform and standard conditions of temperature and humidity and light/dark cycle (12 hr/12 hr) and fed with standard rat chow pellets and tap water ad libitum. All the experiments were performed in accordance with Ethics Committee guidelines for research on laboratory animals of Isfahan University of Medical Sciences, Isfahan, Iran.

***Experimental design***

The animals were randomly assigned into eight normal and eight diabetic groups, 6 rats in each. Vehicle (distilled water containing carboxy methyl cellulose (CMC) 1%) was administered orally (5 ml/kg) in both normal and diabetic control groups. Glibenclamide (Tehran Chemi Co., Tehran, Iran), as reference drug was administered at two doses of 1 and 3 mg/kg orally in normal and diabetic groups respectively.

Other six groups in each normal and diabetic sets were treated with three increasing doses of C. sativus hydroalcoholic (CSHE, 0.2, 0.4, 0.8 g/kg) and three increasing doses of buthnolic extract (CSBE, 0.2, 0.4, 0.8 g/kg). All the treatments with freshly prepared extract as suspensions in CMC 1% were made orally (p.o.) using feeding tube and started 72 hr after diabetes induction. 

***Diabetes induction and blood sampling***

®^22^). Blood samples were taken at 0, 1, 2, 3, 4, 8 hr and the day 9 (216 hr) after treatments using micro-hematocrit capillary tubes (Hirschmann, Germany) and through choroid plexus puncture near the eyes under light ether anesthesia (23®^24^). 

***Statistical analysis***

The measured values were presented as mean±SD. For assessment of difference between groups one-way ANOVA test using SPSS 10 software followed by Bonferroni post hoc test was used. For body weight changes, paired sample t-test was used to compare the means. The results were considered to be significant when the P-values were <0.05. 

## Results

Phytochemical screening tests revealed that hydroalcoholic extract contained alkaloids, steroids, flavanoids, tannins and saponins while anthraquinones were absent. In buthanolic extract on the other hand, flavonoids, saponins and steroids were found but alkaloids, anthraquinones and tannins were not detectable. 

STZ administered i.p., induced diabetes in male Wistar rats after 3 days (Table 2). The body weight of normal and diabetic rats are summarized in Table 1. The final body weight was significantly (*P*< 0.001) decreased in STZ-control group when compared with normal control group. The observed data showed an improvement in body weight after treatment with different doses of CSHE and 

**Table 1.** Changes in body weight of normal, STZ diabetic and orally treated diabetic rats with CSHE and CSBE.

Groups	Initial body weight (g) zero time	Final body weight (g) 9th day	
Normal Ctrl.	212±14	225±11	
STZ Ctrl.	221±10	162 ± 13***
STZ + CSHE 0.2	215±13	195±13^++^	
STZ + CSHE 0.4	223±12	191±11^++^	
STZ + CSHE 0.8	218±14	206±16^+++^	
STZ + CSBE 0.2	236±13	198±11^++^	
STZ + CSBE 0.4	221±19	189±12^++^	
STZ + CSBE 0.8	233±17	211±12^+++^	
STZ + Gliben. 3	207±7	192±10^+^	

Data has been shown as mean±SD. n= 6; *** *P*< 0.001 denotes significant difference compared to normal control (Normal Ctrl.) group.^ + ^*P*< 0.05, ^++ ^*P*< 0.01, ^+++^ *P*< 0.01 denotes significant difference compared

CSBE as well as glibenclamide (3 mg/kg) with respect to the STZ control group (at least *P*< 0.05) (Table 1). As shown in Tables 2& 3, increasing doses of CSHE and CSBE were not effective (*P*> 0.05) in lowering BGLs in the normal or STZ-induced diabetic rats during the first day of treatments (acute phase) compared to controls. On the other hands, both seeds extracts (CSHE; 22.5-33.8 %, CSBE; 26.6-45.0 % decline versus controls) were effective in reducing blood glucose in diabetic rats after prolonged treatments (sub-acute phase) for 8 days irrespective of the doses used (*P*< 0.001) (Table 3). Similar effect was not found by applying the extracts in normal groups (Table 2). 

Glibenclamide, as the reference drug was effective in decreasing BGL in normal (27.8-31.0 % with the dose of 1mg/kg) and diabetic rats (36.0-50.0 % with the dose of 3 mg/kg) and the peak effect was obtained after 3 hr of administration and maintained for at least one hour (Tables 2, 3).

**Table 2.** Effect of orally administered CSHE and CSBE on blood glucose levels (mg/dl) of normal rats.

Time (hr)	0	1	2	3	4	8	216
Groups							
Neg. Ctrl.	86±16	89±13	88±14	90±11	86±16	82±20	87±17
CSHE 0.2	79±23	86±28	91±26	86±21	85±17	79±20	76±20
CSHE 0.4	88±6	97±13	100±14	98±15	99±11	89±15	84±4
CSHE 0.8	75±14	96±11	94±12	94±13	95±22	71±14	70±6
CSBE 0.2	78±20	81±15	79±10	86±16	87±12	78± 22	76±15
CSBE 0.4	80±18	87±10	82±12	82±7	82±8	75±13	80±14
CSBE 0.8	76±24	88±21	84±18	82±17	78±17	78±20	79±10
Gliben. 1	98±14	77±23	64±10*	65±11*	61±8*	71±12	60±7*

Data has been shown as mean±SD. n= 6; * *P*< 0.05 denotes significant difference compared to negative control (Neg. Ctrl.). One-way ANOVA followed by *post hoc* Bonferroni test, CSHE: *Cucumis sativus* hydroalcoholic extract (g/kg), CSBE: *Cucumis sativus* buthanolic extract (g/kg), Gliben.: Glibenclamide (3 mg/kg).

**Table 3.** Effect of orally administered CSE and CSB on blood glucose levels (mg/dl) of STZ-induced diabetic rats.

Time (hr)	0	1	2	3	4	8	216
Groups							
Neg. Ctrl.	321±33	326±30	325±32	323±31	319±28	318±27	320±29
CSHE 0.2	319±54	330±55	339±49	336±78	336±49	323±61	248±38**
CSHE 0.4	301±85	320±83	320±99	306±48	305±88	307±69	234±28***
CSHE 0.8	332±49	349±54	339±57	337±20	335±46	285±10	212±22***
CSBE 0.2	289±25	318±26	307±22	292±24	297±35	297±15	235±29***
CSBE 0.4	283±42	305±31	304±30	293±46	285±44	274±43	178±29***
CSBE 0.8	313±44	328±45	337±48	320±54	326±47	305±44	176±17***
Gliben. 3	317±42	248±42	238±43	207±46**	216±45*	221±50	160±16***

Data has been shown as mean±SD. n= 6; * *P*< 0.05, ** *P*< 0.01, *** *P*< 0.001 denote significant difference compared to negative control (Neg. Ctrl.). One-way ANOVA followed by *post hoc* Bonferroni test,CSHE: *Cucumis sativus* hydroalcoholic extract (g/kg), CSBE: *Cucumis sativus* buthanolic extract (g/kg), Gliben.: Glibenclamide (3 mg/kg)

## Discussion

*C. sativus* seed has been found as a suitable functional food for medical purposes such as diabetes, hyperlipidemia, hypertension (as diuretic), gall bladder stones, constipation and dyspepsia in Asian Traditional Medicine (7, 16-18). In our study, saponin and steroid rich fraction (buthanolic extract) was used in addition to hydroalcoholic total extract to ascertain the active more non-polar ingredients be involved in pharmacologic actions. Regarding to the results, it was found that none of the fractions were effective to cause hypoglycemia in normal groups even after prolonged treatment during sub-acute phase of the study *i.e. *there was no sulfonylurea (*e.g.* glibenclamide) like effects detectable. Our findings are in accordance with Chandrasekar *et al* who investigated blood glucose lowering potential of eight plants of Cucurbitacea family including *C. sativus* fruit extract (20). They revealed that applied fruit ethanolic extract failed to lower blood sugar or to depress the peak value after glucose load intake when administered as a single oral dose of 250 mg/kg to normal and hyperglycemic rats. On the contrary, in our study, aqueous fruit extract of *C. trigouns *Roxb. was able to increase serum insulin level and to decrease the BGL of normal and STZ-induced diabetic rats (25). It had also some beneficial effects in improving the lipid profile of STZ-induced diabetic rats. On the other hand; our results showed that both applied extracts were effective in lowering blood glucose in diabetic animals during the sub-acute phase of the study while there was no significant effect during the first day of the treatment. It was also true for controlling the body weight loss in groups treated with different doses of used extracts which confirmed the anti-diabetic properties of the test extracts at the end of treatment course. Moreover, our findings indicated that the extracts efficacy during sub-acute period of treatment was not dependent on doses used. As a mechanism of action for *C. sativus* seeds in diabetic condition, biguanides like effects (euglycemic action) are supposed (26). Biguanides (*e.g.* metformin) have limited efficacy to lower hyperglycemia and exert their effects by decreasing carbohydrate absorption from the intestine, hepatic gluconeogenesis and glucagon secretion from the pancreas. Increased glycolysis and peripheral sensitivity to insulin are other mechanisms might be accounted for glucose lowering effects of agents with some similarities to biguanides (27). However, measuring the blood insulin and glucagon concentrations in treated rats helps to elucidate more accurately the involved mechanisms. In accordance with our findings; aqueous extract of *Mamordica charantina* L. did not affect the BGLs in normal mice so the investigators concluded that action was derived at least in part, from a decrease in peripheral insulin resistance (12). Based on our findings, it can not be assumed that the active components like saponins, flavons and tannins etc. (7, 14,15) with blood glucose lowering properties were concentrated in buthanolic extract because there was no superiority for this fraction compared with total extract fraction. On the other hand, longer time of administration during sub-acute phase was probably enough to accentuate the delayed mechanisms exerted by two plant seeds fractions. It can be assumed that performing the study during sub-acute period provides a condition in which probable delayed and prolonged pharmacological actions including those related to active metabolites and/or metabolic mechanisms of actions of candidate drug can be revealed. This can support the notion that the metabolic time-consuming mechanisms are mediated by euglycemic agents (26). Further basic and clinical studies are warranted to clarify the involved mechanisms and to indentify the indications for this functional food in metabolic disorders.

## Conclusion

We suggest that *C. sativus* seeds extracts (hydroalcoholic and buthanolic) have some beneficial effects in diabetes control probably through a mechanism similar to euglycemic agents. This claim demands further research to isolate the principal active compounds. The present study was a preliminary investigation and more studies are strongly recommended to clarify the mechanisms involved and the exact role of this herbal medicine in control of metabolic disorders. 
